# The distally based lateral sural neuro-lesser saphenous veno-fasciocutaneous flap: anatomical basis and clinical applications

**DOI:** 10.1007/s10195-012-0202-2

**Published:** 2012-06-26

**Authors:** Chen Wang, Zhuyou Xiong, Jing Xu, Li Zhang, He Huang, Guangzao Li

**Affiliations:** 1 Department of Plastic and Reconstructive Surgery, Shanghai 9th People’s Hospital, Shanghai Jiao Tong University School of Medicine, 639 Zhi Zao Ju Road, Shanghai, 200011 People’s Republic of China; 2 Department of Plastic Surgery, 1st Affiliated Hospital of Bengbu Medical College, 287 Chang Huai Road, Bengbu, 233000 Anhui People’s Republic of China

**Keywords:** Lesser saphenous vein, Lateral sural nerve, Distal based, Tissue defects, Clinical application

## Abstract

**Background:**

Soft tissue management around the lower third of the leg and foot presents a considerable challenge to the plastic surgeon. The aim of this research was to investigate the anatomical relationships of artery, nerve, vein and other adjacent structures in the posterolateral region of the calf, and our experience with using a distally based island flap pedicled with the lateral sural nerve and the lesser saphenous vein for soft tissue reconstruction of lower third of leg, foot, and ankle defects in 15 patients.

**Materials and methods:**

Five fresh cadavers (ten lower limbs) were infused with colored red latex. The origin of the nutrient vessel of the lesser saphenous vein and the lateral sural nerve was identified. Based on the anatomical studies, an island flap supplied by the vascular axis of the lesser saphenous vein and the lateral sural nerve was designed for clinical reparative applications in 15 cases.

**Results:**

The nutrient vessel of the lesser saphenous vein and the lateral sural nerve originates from the superficial sural artery, musculocutaneous perforators of the posterior tibial artery, and septocutaneous perforators of the peroneal artery in different segment of the calf. Meanwhile, these vessels have many sub-branches nourishing subcutaneous tissue and skin, form a favorable vascular chain around the nerve and the vein, and also communicate with vascular plexus of superficial and deep fascia. Among 15 flaps, 13 showed complete survival (86.66 %), while marginal flap necrosis occurred in one patient (6.67 %) and distal wound dehiscence in another (6.67 %). Their appearance and function were satisfactory, with feeling maintained in the heel and lateral side of the foot.

**Conclusions:**

The distally based flap pedicled with the lateral sural nerve and lesser saphenous vein was a reliable source for repairing soft tissue defects in the lower leg and foot due to its advantages of infection control, high survival rate, and sufficient blood supply without the need to sacrifice a major blood vessel.

## Introduction

Reconstruction of soft tissue defects of the foot and the lower third of the leg remains a challenge for trauma and reconstructive surgeons due to poor vascularization and, often, exposure of the calcaneus bone and Achilles tendon. Obtaining weight-bearing repair of these defects requires large flaps with an abundant blood supply and successful anti-infection measures. Ideally, these flaps would fill the dead space and act as a “buffer layer,” accelerating healing. These flaps must be durable, allow a certain range of motion, have a satisfactory shape, retain sensation at the recipient site without loss of the main vessels, and require minimal sacrifice at the donor site. Management options include microsurgical free-flap transfers and pedicled muscle flaps among others, with each method having its own advantages and disadvantages. Masquelet et al. [1] first developed the concept of neurocutaneous island flaps based on the cutaneous branches of the vascular axis around a superficial sensory nerve. The distally based flap pedicled with the vascular plexus surrounding the sural nerve has been widely used to fill lower leg, ankle, or foot defects [2–12]. However, the core gradients of these flaps, the vascular axis of the sural nerve and/or the lesser saphenous vein, were poorly understood. We performed an anatomical study to investigate the origin of the blood supply, the vascular anatomy, the superficial and deep communicating branches between the sural nerve and the lesser saphenous vein, and the feasibility of applying reversed flaps pedicled with the nutrient vessel of the lesser saphenous vein and the lateral sural nerve for the repair of soft tissue defects of the lower leg and foot.

## Materials and methods

### Anatomical study

Five fresh cadavers (ten lower limbs) were injected with red latex through an external iliac artery provided by the Research Center of Clinical Anatomy, Bengbu Medical College. Two horizontal cuts and one vertical cut of the skin were made. The upper horizontal cut was made at the level of the popliteal fossa and the inferior was made at the level of the lateral malleolus. The vertical cut extended from the level of the capitulum fibulae to the level of the posterior margin of the lateral malleolus. After that, the skin, subcutaneous tissue, and fascia were carefully removed and the lesser saphenous vein, sural nerve, and its accompanying artery were identified. The vascular anatomy of the vascular plexus around the sural nerve, the accompanying arteries of the lesser saphenous vein, and the lower peroneal septocutaneous perforators and their communicating branches were examined in detail. Photographs were taken to document the results. All lengths and diameters were measured by a standard rule (precision and accuracy, 0.1 mm) and vernier calipers (precision and accuracy, 0.001 mm), and expressed as (*x* ± *s*) mm (min–max).

### Clinical case series

Fifteen consecutive patients were treated with a distally based flap pedicled with the lateral sural nerve and the lesser saphenous vein from May 2005 and July 2008. All of the patients gave their informed consent prior to being included in the study, and the study was authorized by the local ethical committee and performed in accordance with the ethical standards of the 1964 Declaration of Helsinki as revised in 2000. Twelve patients were men and three were women, with their ages ranging from 14 to 67 years old, and an average age of 44.1 years. Flaps were used to repair defects over the heel, lateral malleolus, and lower leg resulting from crush or avulsion injuries in seven patients, ulcers in three patients, and resection of tumors [including recurrent dermatofibrosarcoma (DFS), recurrent angioblastoma, fibrosarcoma, and recurrent malignant fibrous histiocytoma (MFH)] in four patients. One defect resulted from vascular malformation of the dorsum of the foot. The constructed defects were located in the posterior heel in seven patients, the lateral malleolus in two, the lower leg in fve, and the dorsum of the foot in one. Flap size ranged from 7 × 6 cm to 18 × 13 cm, and the average flap size was 13 × 9.5. The distal pivot point was located 5–9 cm above the lateral malleolus. Table 1 summarizes the data for these patients.

**Table 1:** Summary of patient data

Patient	Sex/age	Cause of defect	Defect site	Defect size (cm)	Flap size (cm)	Flap transport	Pivot point above lateral malleolus tip (cm)	Comorbidity	Results	Complications	Donor site closure	Follow-up (months)
1	M/41	Avulsion injury	Heel	10 × 8	11 × 9	Skin incision	6.5	None	Complete survival		Primary suture	5
2	M/61	Ulcer	Lateral malleolus	14 × 6	16 × 7.5	Skin incision	9	Diabetes	Complete survival		Skin graft	3
3	M/36	Crush injury	Heel	8 × 5	9 × 6	Skin incision	7	None	Complete survival	Venous congestion	Primary suture	6
4	M/22	Avulsion injury	Heel	12 × 8	13 × 9	Skin incision	7.5		Complete survival		Skin graft	9
5	F/63	Avulsion injury	Heel	15 × 10	16 × 11	Skin incision	8				Skin graft	12
6	M/67	Recurrent DFS^a^	Lower leg	12 × 10	14 × 11	Skin incision	6	Smoker	Complete survival	Tension blisters	Skin graft	10
7	M/67	Chronic ulcer	Ankle	12 × 9	13 × 10	Tunnel	8.5		Partial necrosis	Venous congestion	Skin graft	9
8	M/42	Recurrent angioblastoma	Lower leg	17 × 12	18 × 13	Tunnel	7		Complete survival		Skin graft	8
9	M/27	Ulcer	Heel	6 × 5	7 × 6	Skin incision	5.5	None	Complete survival	Tension blisters	Primary suture	11
10	F/14	Traffic accident	Lateral malleolus	11 × 7	12.5 × 8	Skin incision	5		Complete survival		Skin graft	3
11	M/47	Crush injury	Lower leg	16 × 9.5	18 × 10.5	Skin incision	7.5		Complete survival		Primary suture	16
12	M/45	Fibrosarcoma	Lower leg	14 × 11	15 × 12	Tunnel	7	None	Complete survival		Skin graft	24
13	F/34	Vascular malformation	Dorsum of foot	13 × 9	14 × 10	Skin incision	8.5	Hepatitis B	Distal wound dehiscence	Infection	Skin graft	4
14	M/64	Recurrent MFH^b^	Lower leg	13 × 10	15 × 11	Tunnel	6	Diabetes	Complete survival		Skin graft	12
15	M/31	Crush injury	Heel	9 × 7	10 × 8	Skin incision	6.5		Complete survival		Primary suture	15

^a^Dermatofibrosarcoma

^b^Malignant fibrous histiocytoma

### Surgical technique

All patients underwent preoperative evaluations including clinical evaluation of peripheral pulses and perfusion of skin. With the patient in the prone position, a preoperative Doppler probe was used in all cases to spot the perforators of the peroneal artery in the area posterior and proximal to the lateral malleolus, with the largest perforator spot used as a pivot point. An axial line was drawn from the midpopliteal point to the midpoint between the Achilles tendon and the lateral malleolus.

The procedures were performed when patients were under general or continuous epidural anesthesia. The patients were positioned in a lateral decubitus position. Before inflating the tourniquet, the tumor was excised widely or the wound was debrided and irrigated. The pattern of the recipient site was used to determine the dimensions and design of the flap. The shape of the flap depended on the size of the defect to cover. Usually, the distal part of the flap was tailored into a teardrop configuration to facilitate the closure of the skin over the pedicle without tension. The lateral sural nerve, superficial sural artery, and lesser saphenous vein were included in the flap. A 2–3 cm wide strip pedicle was adopted here, as such a design not only protects the lesser saphenous vein and the small vessel in the pedicle but it also acts as an index of distraction tension in the rotating flap. The flap was then raised from the subfascial plane in a proximal-to-distal direction. The lateral sural nerve and lesser saphenous vein were ligated proximally to the flap. When the confluence point of the medial/lateral sural nerves was high, a pedicle microdissection was necessary to separate the medial sural nerve from the pedicle. Once the skin and fascia were elevated as a unit, dissection was carried out distally until the pivot point was reached. A skin incision was opened to communicate the area just above the pivot point with the proximal aspect of the defect to be covered. The tourniquet was deflated, and the circulation in the flap was checked. The flap was then transposed distally and sutured to the receptor site. The opened skin bridge was partially covered with the extension of the flap to decrease the pressure over the pedicle. The donor area was either primarily closed or skin grafted, depending on the dimensions. A well-padded dressing and plaster fixation were applied to keep the ankle in the neutral position for 2–3 weeks.

## Results

### Anatomical study

**Fig. 1:**
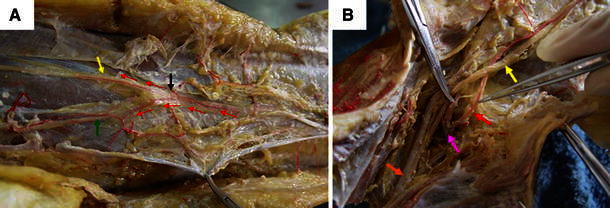
**a** The red-latex-injected dissected specimen, showing the lateral sural nerve (*yellow arrow*), the medial sural nerve (*green arrow*), with the vascular plexus around them (*red arrows*). The *black arrow* indicates the convergence point of the medial and lateral sural nerves. **b** The injected dissected specimen, showing the superficial sural artery (*red arrow*), which originates from the peroneal artery (*pink arrow*), accompanied by the lateral sural nerve (*yellow arrow*) arising from the common peroneal nerve (*orange arrow*) in the initial segment at the midpoint of the leg (color figure online)

The medial sural nerve and lateral sural nerve, which originate from the posterior tibial nerve and the common peroneal nerve, respectively, travel along the surface of the gastrocnemius muscle, which pierces the deep fascia, and descend gradually toward the middle line, forming the sural nerve together. The convergence point of these nerves is most commonly located at the lower third of the leg, about 8.5 ± 0.8 mm (range 2.5–15.0 mm) above the tip of the lateral malleolus (Fig. 1a). There are communicating branches between these two nerves in four out of ten cases.

The superficial sural artery was found to originate from the peroneal artery, with an outer diameter of 1.08 ± 0.14 mm (range 0.9–1.4 mm) at the jumping-off point. Accompanying the lateral sural nerve in the initial segment, it pierces the fascia at the level of the middle third and upper third of the lower leg junction, and travels with the lesser saphenous vein, sending out several branches along its course (to the medial/lateral sural nerve, lesser saphenous vein, deep fascia, and subcutaneous fat layer) in the upper segment of the posterior-lateral leg (Fig. 1b).

**Fig. 2:**
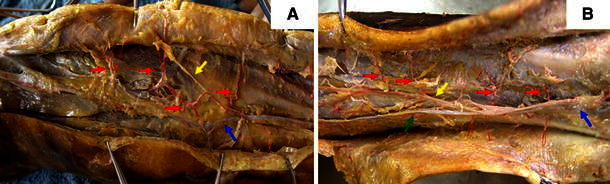
**a** Dissected specimen showing the musculocutaneous perforators (*red arrows*) originating from the posterior tibial artery, which are seen to branch to the lateral sural nerve (*yellow arrow*), the lesser saphenous vein (*blue arrow*), and the subcutaneous layer at the mid-point of the leg. **b** Dissected injected specimen showing intermuscular septum perforators (*red arrows*) of the peroneal artery below the mid-point of the leg, with the distribution of perforators to the lateral sural nerve (*yellow arrow*), the medial sural nerve (*green arrow*), the lesser saphenous vein (*blue arrow*), and the subcutaneous layer (color figure online)

There were 3–6 musculocutaneous perforators originating from the posterior tibial artery at the midway of the lower leg, with an outer diameter adjacent to the gastrocnemius muscle of 0.56 ± 0.07 mm (range 0.3–1.0 mm). The perforators gave off numerous branches that were distributed to the medial/lateral sural nerve, lesser saphenous vein, deep fascia, and subcutaneous fat layer (Fig. 2a). Also, at the midpoint of the lower leg, the nerve-artery complex becomes suprafascial and is accompanied by the lesser saphenous vein (Fig. 2a). Four to seven intermuscular septum perforators of the peroneal artery were observed at the lower third of the lower leg, with an outer diameter of 0.58 ± 0.06 mm (range 0.2–1.1 mm), that were distributed to the sural nerve, lesser saphenous vein, deep fascia, and subcutaneous fat layer (Fig. 2b). In the lateral and posterior lower leg, the above three arteries send out branches that communicate with each other and form a three-dimensional vascular plexus around the medial/lateral sural nerve and lesser saphenous vein in the layer of deep fascia.

**Fig. 3:**
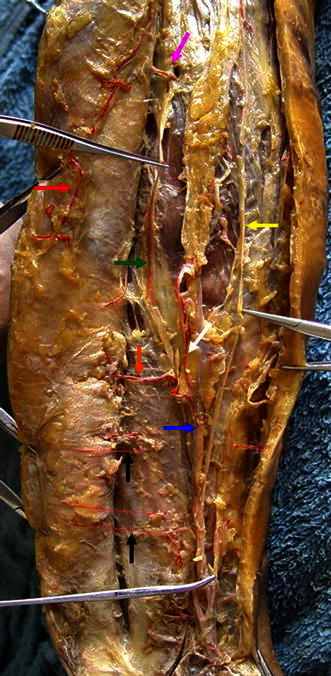
Dissected injected specimen showing the suprafascial course of the medial sural nerve (*green arrow*), the lateral sural nerve (*yellow arrow*), and the lesser saphenous vein (*blue arrow*) along with the surrounding vascular plexus. The *red arrow* indicates the superficial sural artery, the *pink arrow* indicates the musculocutaneous perforators of the posterior tibial artery, and the *orange arrow* indicates the peroneal artery. The *white arrow* shows the contribution of the accompanying artery of the lesser saphenous vein to the plexus around the medial/lateral sural nerve, which also anastomoses with the communicating branch from the deep fasia and the subcutaneous vessel networks (*black arrow*) (color figure online)

In conclusion, during the course of the medial/lateral sural nerve and the lesser saphenous vein, there are accompanying arteries with relatively constant outer diameters around them, which anastomose with the superficial sural artery, musculocutaneous perforators of the posterior tibial artery, and septocutaneous perforators of the peroneal artery in different segments of the calf, forming a fine interlacing vascular plexus around the nerve and the vein. This vascular plexus also anastomoses with the communicating branch from the deep fascia and the subcutaneous vessel networks, as described previously (Fig. 3).

### Clinical case series

Thirteen flaps (86.66 %) survived completely. One patient had skin necrosis reaching approximately 1 cm^2^ at the distal margin of the flap which required a split-thickness skin graft for wound closure after carefully debridement. Another patient developed distal wound dehiscence which healed well after excision of the wound margins. All other flaps healed well after three weeks of dressing, as summarized in Table 1. The donor site healed adequately in all patients. In 11 cases, the donor site was closed with a split-thickness skin graft, while the other four cases were closed primarily. There was no complaint of a loss of sensation along the lateral border of the foot and heel. The average follow-up was 11 months, ranging from 3 to 24 months. No symptomatic neuromas occurred. No recurrent ulceration was found in the weight-bearing areas. All patients were able to bear weight on the operated extremity throughout the duration of follow-up.

### Case 3

**Fig. 4a–d:**
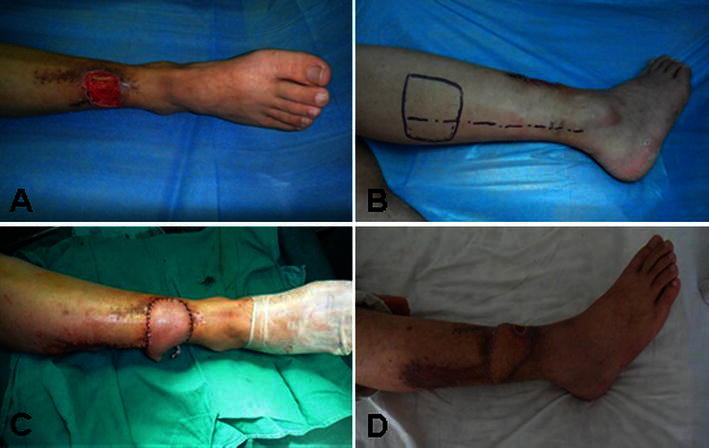
Case 3. **a** A 36-year-old male suffered an accidental injury to the anterior tibia of his right leg. Skin defect of the anterior tibia and exposure of bone following numerous debridements are shown. **b** A distally based neuro-veno-fasciocutaneous island flap was designed. **c** The defect was covered with the island flap, and the donor site was skin-grafted. **d** Both the flap and donor site healed completely, presenting satisfactory condition at follow-up

A 36-year-old man was hospitalized due to a traffic accident and injury to his leg sustained one month previously. Following debridement, a skin defect of the right anterior tibia remained, measuring 8.0 × 5.0 cm, with bone exposure (Fig. 4a). A distally based neuro-veno-fasciocutaneous island flap was designed to cover the defect, measuring 9.0 × 6.0 cm (Fig. 4b). The pivot point of the flap was located 7.0 cm proximal to the tip of the malleolus, with a pedicle width of 3.5 cm. The lesser saphenous vein and the lateral sural nerve were ligated, the island flap was transferred to the defect, the area was fixed with plaster, and the donor site was skin-grafted (Fig. 4c). Postoperatively, the flap survived with good vascular supply, and the wound and donor site healed completely (Fig. 4d).

### Case 8

**Fig. 5a–f:**
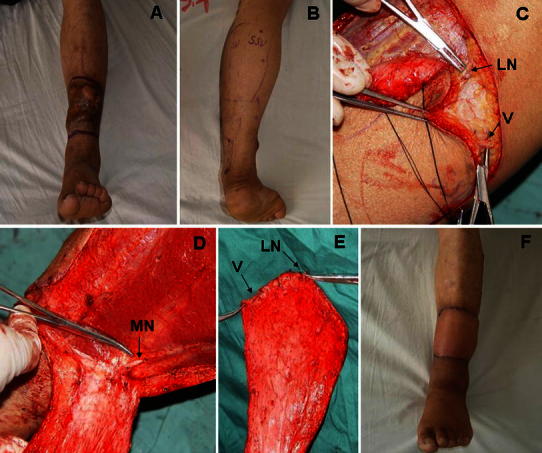
Case 8. **a** An ulcer on the lower left leg caused by recurrent angioblastoma. **b** A distally based island flap pedicled with the lateral sural nerve and lesser (short) saphenous vein was planned. **c** The island flap was harvested with the lateral sural nerve and lesser saphenous vein. *LN* lateral sural nerve, *V* lesser saphenous vein. **d** Meticulous dissection from deep fascia preserved the medial sural nerve (MN). **e** The flap pedicle contains the lateral sural nerve and lesser saphenous vein, which were cut and ligated at the distal ends. *LN* lateral sural nerve, *V* lesser saphenous vein. **f** The flap survived completely, displaying a good appearance

A 42-year-old man suffered from recurrent angioblastoma over the anterior tibial skin of his left leg, and formed an ulcer on the surface (Fig. 5a). After excision, there was a 17 × 12 cm defect on the lower left leg, and there was exposure of the anterior tibial muscle and bone. A distally based island flap was outlined at the junction of the two heads of the gastrocnemius muscle. A meticulous dissection was traced over the presumed course of the lateral sural nerve and short saphenous vein from deep fascia, the medial sural nerve was preserved, and the lateral sural nerve and lesser saphenous vein were cut and ligated at the distal ends (Fig. 5e). The wound was covered with a distally based neuro-veno-fasciocutaneous island flap measuring 18.0 × 13.0 cm, with a 8.0 × 3 cm pedicle. The rotation point of the island flap was located 7.0 cm proximal to the tip of the malleolus. The flap was then sutured to the receptor site after a 90° rotation. A skin graft was placed at the donor site at the same time. The flap healed uneventfully with a soft texture and a similar color to the surrounding skin (Fig. 5f).

## Discussion

Ever since Ponten first reported the fasciocutaneous flap from the lower leg in [13], the vascularization of the sural region of the lower leg has been extensively investigated, and the concepts of neurocutaneous, neuro-veno-fasciocutaneous, and fasciomusculocutaneous flaps have been realized [1, 12, 14–18]. Nakajima et al. [19] described that accompanying arteries of the lesser saphenous vein and sural nerve gave off venocutaneous and neurocutaneous perforators which nourish the skin from the calf down to the ankle. Based on this concept, Nakajima et al. [14] considered that raised flaps were based only on the circulation from the accompanying artery of the lesser saphenous vein, thus preserving the sural nerve and the sensation along the lateral border of the foot. Anatomical studies showed that the major blood supplies of neurocutaneous flaps are the segmental arteries of the cutaneous nerve along the cutaneous nerve trunk [6, 20]. Two intraneural and paraneural vascular networks with a vertical chain-like anastomosis guarantee a long-distance supply [21]. The distally based superficial sural artery flap is one of these neurocutaneous flaps, and its circulation depends on the anastomosis of the perisural nerve vasculature with distal perforators of the peroneal artery near the lateral malleolus. Zhang et al. [22] demonstrated that there are two kinds of nutrient vessels of the lesser saphenous vein (the nutrient vessels of nerve–vein and vein–nerve) which constitute the para-vein vascular trunk and the vein–wall vascular plexus of the lesser saphenous vein. In this study, we identified the arterial anatomy of the upper lateral leg and found that the superficial sural artery was the major supply vessel. In addition, 3–5 musculocutaneous perforators of the posterior tibial artery were identified at the middle one-third of the leg, with the biggest giving off several branches to bridge the perforators of the peroneal artery. In the lower half of the leg, we observed the intermuscular branches of the peroneal artery along with the largest terminal branch of the peroneal artery at the lateral supramalleolar. These findings provided the vascular basis for flap design. Distally based compound flaps (including musculocutaneous, fasciomusculocutaneous, osteocutaneous, and myo-osteocutaneous flaps) of the sural nerve and lesser saphenous vein have been used for the repair and coverage of lower leg ulcers, osteomyelitis, bone exposures, and exposed internal hardware [6, 12].

In our research, the distally-based sural neuro-veno-fasciocutaneous flap was pedicled with the nutrient arteries of the lateral sural nerve and the lesser saphenous vein originating from the superficial sural artery and the musculocutaneous perforators of the posterior tibial artery as well as the interseptum perforators of the downward peroneal artery. These formed chain-linked vascular plexuses by connecting with each other and the anastomosis of the vascular networks from the superficial fascia, deep fascia and subdermis. There are abundant communicating branches between these vascular networks, meaning that the arteries to nerves, veins, fascia, and skin share a common origin, thus forming a multisegmental vascular plexus along the whole nerve trunk with ample blood supply. This provides abundant blood perfusion for the flap.

Nakajima et al. [14] proposed a pedicle design that included the lesser saphenous vein, based on research into the peripheral vascular network of the limbs. They found in their study that the lesser saphenous vein in the flap not only improves the venous outflow and the circulation of the flap, but it also allows cranial extension of the flap over the proximal third of the calf. The flap size depended on where it was raised in clinical practice. The nutrient vessels of the lesser saphenous vein and the lateral sural nerve show a close anastomosis with the perforators in surrounding fascias, which leads to an affluent and multidimensional vascular network in the lower leg, as demonstrated by our study. Skin areas supplied by extraterritorial flow were elusive. Therefore, it was a risk to pursue a larger flap blindly, whereas protecting the perforator or terminal branch of the peroneal artery over the lateral malleolus and bringing it into the pedicle was a safer approach in the procedure.

Whether to perform caudal ligation of the lesser saphenous vein or not is a prominent concern in the literature [3, 15]. Some researchers insist that lesser saphenous ligation or anastomosis with a draining vein proximal to the recipient area could help to reduce the burden on venous drainage, thus alleviating congestion of the flap [23]. However, in our opinion, such a ligation would destroy the vascular plexus and further affect the survival of the flap adversely. Meanwhile, as we know, a distally based neuro-veno-fasciocutaneous flap does not have high arterial blood perfusion, and reverse flow cannot occur in the large superficial vein [3]. In addition, our anatomical investigation verified that numerous small, long veins that run between the lesser saphenous vein and the posterior tibial vein at the posterolateral malleolus are important channels for venous drainage. Based on these findings, the lesser saphenous veins were all preserved in the pedicle rather than ligated in our study. During the follow-up visit, none of those patients had suffered from venous congestion and permanent foot swelling. Moreover, hyperbaric oxygen therapy (HBOT) was employed postoperatively for flaps suffering from edema, stasis, and cyanosis. This is a good method for preventing venous congestion, tension blisters, and some infections [12].

Jeng et al. [16] described a sensory sural island flap including the sural nerve and inosculated recipient nerves. Recently, sensory function was established of a skin flap and foot via end-to-side neurorrhaphy between the sural nerve and the superficial peroneal nerve or its branches [24–26]. However, a disadvantage of sensation reconstruction is the partial or complete necrosis that occurs after the operation, due to possible damage to the perforators and the peripheral vessel networks when isolating. In the present study, we had the advantage that the medial sural nerve was retained, as no one lost feeling in either the foot or the flap, which gradually innervated postoperation.

In conclusion, the distally based island flap pedicled with the nutrient vessels of the lesser saphenous vein–lateral sural nerve, including the perforators of the peroneal artery around the ankle region, was a reliable source for covering soft tissue defects in the lower one-third of the leg, ankle, and foot. The procedures only involved a single operation without the need for microsurgical anastomosis, and yielded a more durable and sensate skin cover. It also does not require the sacrifice of a main blood vessel and sensation in the foot. Therefore, this distally based lateral sural neuro-lesser saphenous veno-fasciocutaneous flap should be considered to be a good choice of flap for reconstruction of the lower one third of the leg, foot, and ankle.
